# Robotic, laparoscopic or open hemihepatectomy for giant liver haemangiomas over 10 cm in diameter

**DOI:** 10.1186/s12893-020-00760-5

**Published:** 2020-05-06

**Authors:** Minggen Hu, Kuang Chen, Xuan Zhang, Chenggang Li, Dongda Song, Rong Liu

**Affiliations:** Department of Hepatobiliary and Pancreatic Surgical Oncology, Chinese PLA General Hospital and Chinese PLA Medical School, 28 Fuxing Road, Beijing, 100853 China

**Keywords:** Giant liver haemangioma, Hemihepatectomy, Laparoscopic liver resection, Robotic liver resection, Clinical effects

## Abstract

**Background:**

To evaluate the clinical efficacy of robotic, laparoscopic, and open hemihepatectomy for giant liver haemangiomas.

**Methods:**

From April 2011 to April 2017, consecutive patients who underwent hemihepatectomy for giant liver haemangiomas were included in this study. According to the type of operation, these patients were divided into the robotic hemihepatectomy (RH) group, the laparoscopic hemihepatectomy (LH) group, and the open hemihepatectomy (OH) group. The perioperative and short-term postoperative outcomes were compared among the three groups. The study was reported following the STROCSS criteria.

**Results:**

There were no significant differences in age, sex, tumour location, body surface area (BSA), future liver remnant volume (FLR), standard liver volume (SLV), liver haemangioma volume, FLR/SLV, resected normal liver volume/resected volume, hepatic disease, rates of blood transfusion, liver function after 24 h of surgery, operative morbidity and mortality among the three groups. Compared with patients in the RH group (*n* = 19) and the LH group (*n* = 13), patients in the OH group (*n* = 25) had a significantly longer postoperative hospital stay (*P* < 0.05), time to oral intake (*P* < 0.05), and time to get-out-of-bed (*P* < 0.05); a higher VAS score after 24 h of surgery (*P* < 0.05); and a shorter operative time (*P* < 0.05). There were no significant differences in these postoperative outcomes (*P*>0.05) between the RH group and the LH group. When the setup time in the RH group was excluded, the operative time in the RH group was significantly shorter than that in the LH group (*P*<0.05). There was no significant difference in the operative time between the RH group and the OH group (*P*>0.05). The amount of intraoperative blood loss in the RH group was the lowest among the three groups (*P*<0.05), and the amount of intraoperative blood loss in the LH group was less than that in the OH group (*P*<0.05).

**Conclusion:**

Robotic and laparoscopic hemihepatectomies were associated with less intraoperative blood loss,better postoperative recovery and lower pain score. Compared with laparoscopic hemihepatectomy, robotic hemihepatectomy was associated with significantly less intraoperative blood loss and a shorter operative time.

## Background

Haemangioma is the most common benign lesion of the liver, occurring in the general population with a prevalence that ranges from 0.4 to 7.3% based on autopsy findings [1, 2]. The majority of liver haemangiomas are asymptomatic and are often discovered incidentally during imaging investigations for various unrelated pathologies. Asymptomatic patients with liver haemangiomas less than 5 cm in diameter require observation and no intervention [3]. Surgical treatment for liver haemangioma is required in lesions larger than 5 cm in diameter, when there are symptoms or complications, or when the diagnosis is uncertain [4]. The main treatments for liver haemangioma include transarterial embolization (TAE), enucleation, liver resection, and transplantation. A giant liver haemangioma is defined as a liver haemangioma with a minimum size of 10 cm [5]. While some surgeons have reported that enucleation is safer and quicker than resection [6, 7], others have concluded that there is no significant difference between enucleation and resection [8, 9]. Mark S et al. [10] suggested that liver resection was preferable for lesions that completely occupied an anatomical section of the liver. Open liver resection requires a large abdominal incision and long recovery time. Increasing numbers of operations are now performed with minimally invasive surgery with either laparoscopic or robotic surgery [11]. To our knowledge, no study has been reported that has compared robotic, laparoscopic, and open liver resection for giant liver haemangiomas. The present study was undertaken to evaluate the clinical efficacy of robotic, laparoscopic and open hemihepatectomy for giant liver haemangioma.

## Methods

### Patients

**Fig. 1:**
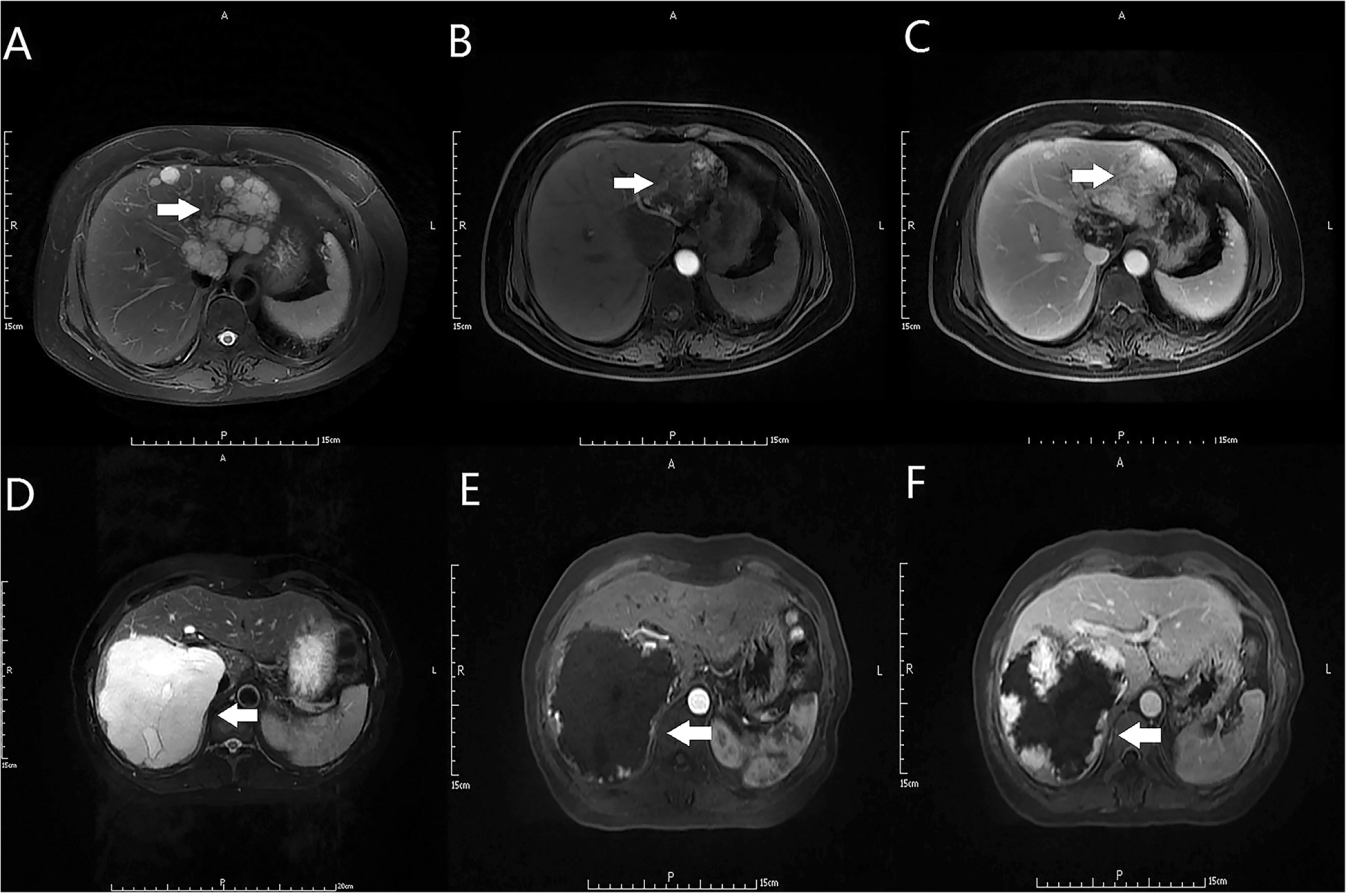
Magnetic resonance imaging of giant liver haemangiomas. **a** and **d** T2-weighted magnetic resonance shows giant liver haemangiomas on the left and right liver (A and D, arrow). **b** and **e** Magnetic resonance in the arterial phase shows giant liver haemangiomas on the left and right liver (B and E, arrow). **c** and **f** Magnetic resonance in the delayed phase shows giant liver haemangiomas on the left and right liver (C and F, arrow)

**Fig. 2:**
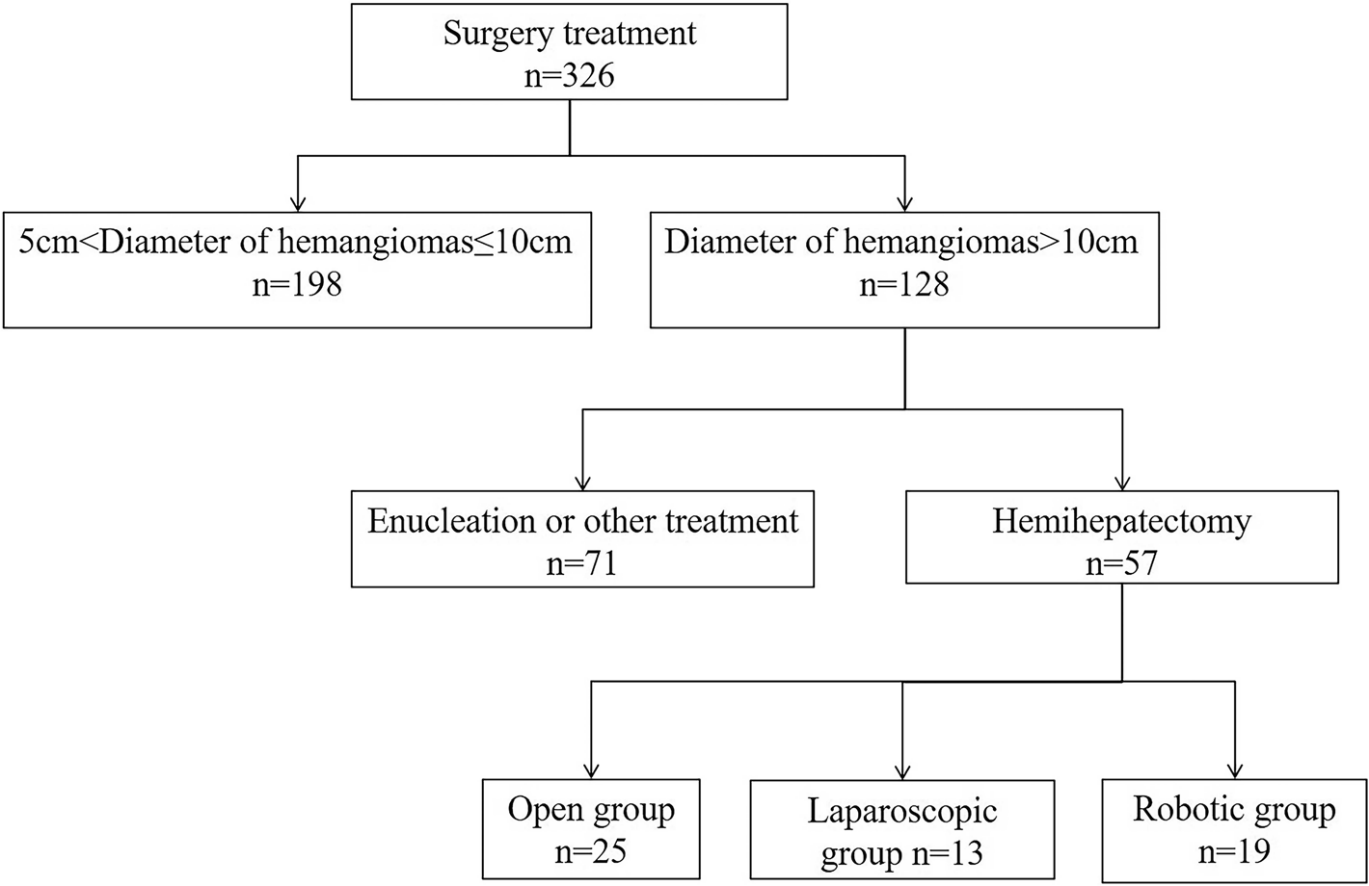
Search strategy

This was a retrospective study on consecutive patients with liver haemangioma who underwent hemihepatectomy from April 2011 to April 2017 in our hospital. The diagnosis of giant liver haemangioma was made by computed tomography and/or magnetic resonance imaging and postoperative histopathology (Fig. 1). The main indication for operation was a giant liver haemangioma (> 10 cm in diameter) with symptoms (abdominal pain, nausea, or premature satiety after a meal). These patients were all suitable for undergoing robotic, laparoscopic and open hemihepatectomy. The choice of operation was determined by the patient after discussion with the operating surgeons. All the operations were carried out by a single team of experienced liver surgeons. The variables selected for analysis were age, sex, tumour size, tumour location, hepatic disease, operative time, intraoperative blood loss, rate of blood transfusion, postoperative hospital stay, time to oral intake, time to get-out-of-bed, liver function after 24 h of surgery, visual analogue scale (VAS) score after 24 h of surgery, and operative morbidity/mortality. According to the type of operation, the patients were divided into the robotic hemihepatectomy (RH) group, laparoscopic hemihepatectomy (LH) group, and open hemihepatectomy (OH) group. Comparisons of the variables were then made among the three groups. The medical history was taken and physical examination and liver ultrasonography were routinely carried out at a follow-up visit 3 months after surgery. The data in our study that came from a single study centre were acquired retrospectively. As we know, potential biases naturally exist in respective studies. However, we developed a rigorous and scientific search strategy to lower the power of this bias (Fig. 2). This study was approved in writing by the Beijing Special Clinical Application Program (Grant No. Z171100001017239 and Grant No. Z151100004015004).

### Measurements of liver volumes

The volumes of the future liver remnant (FLR) and liver haemangioma were calculated based on computed tomographic (CT) volumetry. The CT data were transferred to a workstation for assessment. Liver volumes were calculated by the integrated software technique. A standard liver volume (SLV) was calculated using the following formula: liver volume (cm^3^) = 706 × body surface area (m^2^) + 2.4 [12]. This volume has been validated in a meta-analysis to be a precise and unbiased method to estimate total liver volume [13]. The ratio of FLR volume to total liver volume was estimated using the following formula: FLR/SLV. The body surface area (BSA) was calculated using the following formula: body surface area (m2) = [body weight (kg) × body height (cm) ÷ 3600]^0.5^ [14]. The resected volume was calculated using the following formula: resected volume (cm3) = standard liver volume (cm^3^) - future liver remnant volume (cm^3^). The resected normal liver volume was calculated using the following formula: resected normal liver volume (cm^3^) = resected volume (cm^3^) - liver haemangioma volume (cm^3^). The ratio of resected normal liver volume to resected volume was estimated using the following formula: resected normal liver volume/resected volume.

### Surgical techniques

#### Robotic Hemihepatectomy

**Fig. 3:**
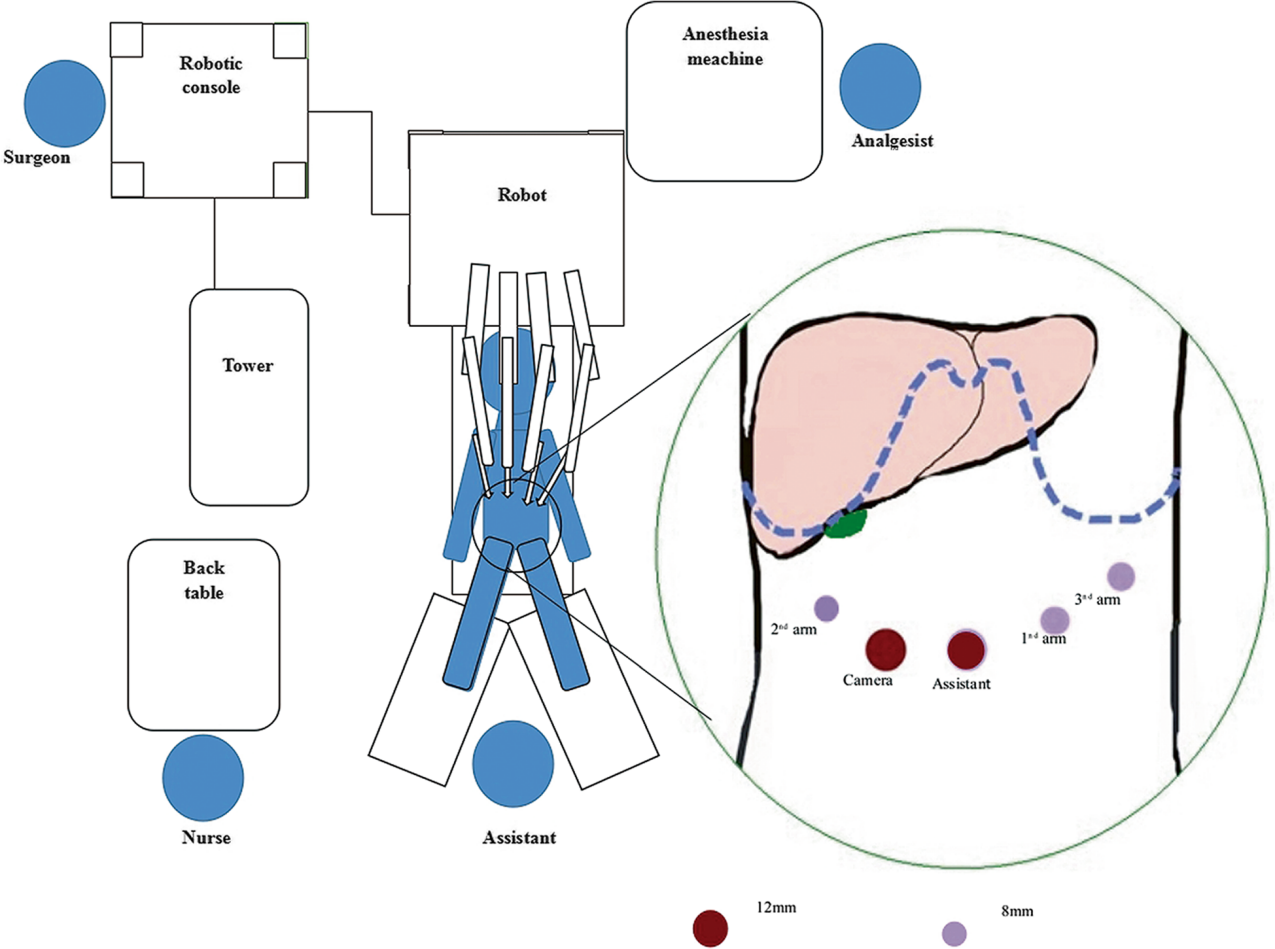
Operating room setup and port placement for robotic right hemihepatectomy

**Fig. 4:**
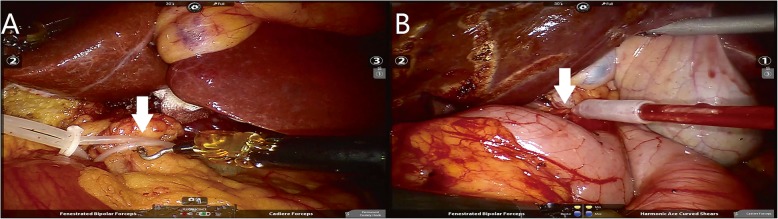
Modified Pringle manoeuvre. **a**, **b** Hepatoduodenal ligament was encircled and ready to be occluded by the catheter (8F) or rope: hepatoduodenal ligament (A and B, arrow)

**Fig. 5:**
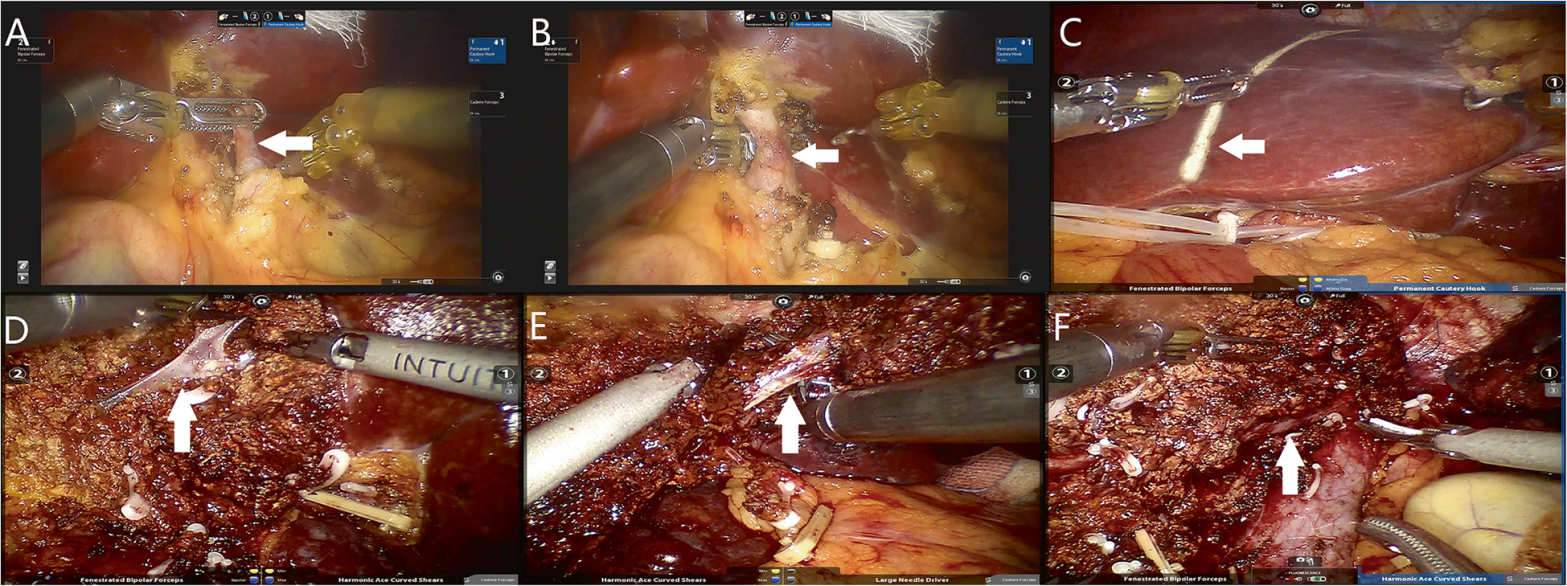
The main procedures of robotic left hemihepatectomy. **a** The left hepatic artery (A, arrow) was dissected and sectioned with clips and an ultrasound scalpel. **b** The left branch of the portal vein (B, arrow) was dissected and sectioned with clips and an ultrasound scalpel. **c** The ischaemic demarcation line (C, arrow) was incised by using a monopolar hook. **d** The branches of the middle hepatic vein (D, arrow) were dissected and sectioned with clips and an ultrasound scalpel. **e** The left hepatic vein was dissected and sectioned by using a linear vascular endo-stapler (E, arrow). **f** Hepatic cross section after hemihepatectomy and the middle hepatic vein (F, arrow)

**Fig. 6:**
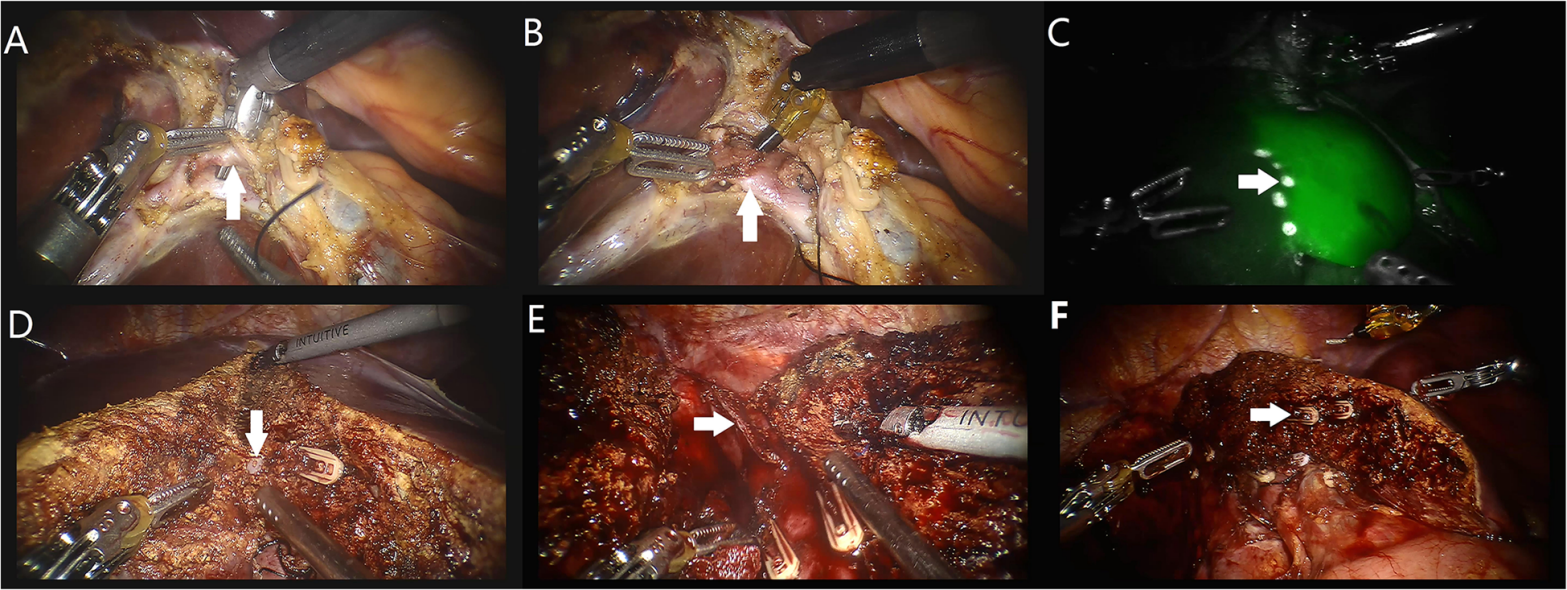
The main procedures of robotic right hemihepatectomy. **a** The right hepatic artery (A, arrow) was dissected and sectioned with clips and an ultrasound scalpel. **b** The right branch of the portal vein (B, arrow) was dissected and sectioned with clips and an ultrasound scalpel. **c** The ischaemic demarcation line (C, arrow) was incised by using a monopolar hook with the application of indocyanine green (ICG) fluorescence imaging. **d** The branches of the middle hepatic vein (D, arrow) were dissected and sectioned with clips and an ultrasound scalpel. **e** The right hepatic vein was dissected and sectioned by using a linear vascular endo-stapler (E, arrow). **f** Hepatic cross section after hemihepatectomy and branches of the middle hepatic vein (F, arrow)

The patient was placed in a modified lithotomy and reverse Trendelenburg position, with the first assistant standing between the patient’s legs. For right hemihepatectomy, after general anaesthesia with endotracheal intubation, the first trocar was inserted at the umbilical site after creating pneumoperitoneum. Intraabdominal pressure was controlled at 12 to 14 mmHg (1 mmHg = 0.133 kPa). The robotic camera was inserted through the umbilical port, and the other four ports were introduced under laparoscopic view. The camera port was then placed in the right paraumbilical area. The first and second robotic arm ports were introduced in the left and right upper quadrant areas, respectively. The umbilical port was used as the assistant’s port. The third robotic arm port was introduced at the left anterior axillary line (Fig. 3). For left hemihepatectomy, port placement was similar to that of right hemihepatectomy, except that the placements of the camera port and the assistant’s port were swapped. Selective hemihepatic inflow occlusion was used. The modified Pringle’s manoeuvre was used to occlude inflow of the entire liver when necessary (Fig. 4). Liver parenchymal transection was performed using an ultrasound scalpel. Intraparenchymal control of major vessels was achieved with clips or sutures (Figs. 5, 6). The corresponding hepatic pedicle and hepatic vein were transected with a linear vascular endo-stapler. The resected specimen was placed in a specimen bag and retrieved from the abdomen through an extension of the umbilical port wound.

#### Laparoscopic Hemihepatectomy

The patient was placed in a supine position with the patient’s legs abducted. The surgeon stood between the patient’s legs, and the first assistant stood on the right side of the patient. After general anaesthesia with endotracheal intubation, the first trocar was inserted into the umbilical site after creating pneumoperitoneum. Intraabdominal pressure was controlled at 12 to 14 mmHg (1 mmHg = 0.133 KPa). Four ports were usually used. The operating ports were placed in a fan shape around the lesion. Selective hemihepatic inflow occlusion was used for hemihepatectomy. The modified Pringle’s manoeuvre was used to occlude the inflow to the entire liver when necessary. Liver parenchymal transection was performed using an ultrasound scalpel. Intraparenchymal control of major vessels was achieved with clips or sutures. The corresponding hepatic pedicle and hepatic vein were transected using a linear vascular endo-stapler. The resected specimen was placed in a specimen bag and retrieved from the abdomen through an extension of the umbilical port wound.

#### Open Hemihepatectomy

The patient was placed in a supine position. After general anaesthesia with endotracheal intubation, laparotomy was carried out via a right subcostal incision. After exploration of the abdominal cavity, hepatic vascular inflow occlusion was similar to the technique used in laparoscopic hemihepatectomy. Liver parenchymal transection was performed using an electrotome or ultrasound scalpel. Haemostasis was achieved with monopolar cautery, sutures, or clips.

### Statistical analysis

Qualitative variables were compared using the chi-square test. The likelihood ratio test was used for data when the theoretical frequency was less than 5. The data are expressed as the mean ± standard deviation. Continuous variables were compared using ANOVA. If ANOVA indicated that there were differences between the three groups, the SNK (Student-Newman-Keuls) test was used for further verification. A *P*-value of < 0.05 was considered to be statistically significant. Statistical analyses were performed using SPSS software (version 19.0, IBM Corp, Armonk, NY). The study was reported following the STROCSS criteria.

## Results

During the study period, 326 patients underwent liver resection for liver haemangioma, and 128 patients had giant liver haemangiomas (defined as a liver haemangioma with a diameter > 10 cm). Hemihepatectomies were carried out in 57 of these patients with giant liver haemangiomas. RH was carried out in 19 patients, LH in 13 patients, and OH in 25 patients. There were no significant differences in age, sex, tumour location, BSA, FLR, SLV, liver haemangioma volume, FLR/SLV, resected normal liver volume/resected volume, or hepatic disease among the three groups (Table 1).

**Table 1:** Characteristics of Patients in the RH ,LH and OH Groups

	*RH group*	*LH group*	*OH group*	
	*n = 19*	*n = 13*	*n = 25*	*P value*
Age (year)	49.2 ± 10.6	46.5 ± 8.9	45.6 ± 10.1	0.494
Sex				
Male	2 (10.5%)	1 (7.7%)	8 (32%)	0.094
Female	17 (89.5%)	12 (92.3%)	17 (68%)	
Location*				
Right	15	8	19	0.530
Left	4	5	6	
Body surface area (BSA, cm2)	1.6 ± 0.2	1.6 ± 0.2	1.6 ± 0.2	0.821
Futrue liver remnant volume (FLR, cm3)	463.9 ± 151.2	442.9 ± 152.8	374.8 ± 135.7	0.115
Standard liver volume (SLV, cm3)	1120.1 ± 137.8	1110.1 ± 108.1	1144.9 ± 108.8	0.646
Liver hemangioma volume (cm3)	553.2 ± 122.3	556.2 ± 179.8	667.5 ± 202.6	0.061
FLR/SLV (%)	41.2 ± 11.4	40.1 ± 14.0	33.2 ± 13.4	0.099
Resected normal liver volume / resected volume (%)	16.1 ± 7.2	17.4 ± 7.0	14.2 ± 8.2	0.437
Hepatic disease†	0 (0%)	0 (0%)	0 (0%)	1.000

*Location refers to the location of the largest liver hemangioma for patients with multiple lesions

†Hepatic disease refers to hepatitis and liver cirrhosis

There were no significant differences in the rates of blood transfusion (*P*>0.05, Table 2) among the three groups. The operative time of the OH group was significantly shorter than that of the RH group and the LH group (190.2 ± 51.8 vs 256.3 ± 57.7 and 268.4 ± 93.6 min, *P*<0.05, Tables 2, 4), while there was no significant difference between the RH group and the LH group (*P*>0.05, Table 4). When the setup time in the RH group was excluded, the operative time of the RH group was significantly shorter than that of the LH group (216.3 ± 57.7 vs 268.4 ± 93.6 min, *P*<0.05, Tables 2, 4), while there was no significant difference between the RH group and the OH group (190.2 ± 51.8 vs 216.3 ± 57.7, *P*>0.05, Table 4). The amount of intraoperative blood loss in the RH group was significantly the lower among the three groups (319.5 ± 206.0 vs 476.9 ± 210.8 and 628.0 ± 231.0 ml, *P*<0.05, Tables 2, 4), and the amount of intraoperative blood loss in the LH group was significantly less than that in the OH group (476.9 ± 210.8 vs 628.0 ± 231.0 ml, *P*<0.05, Tables 2, 4). Compared with patients in the RH group and the LH group, patients in the OH group had a significantly longer postoperative hospital stay (7.2 ± 2.3 vs 5.5 ± 2.1 and 4.7 ± 1.7 d), time to oral intake (3.1 ± 1.1 vs 2.2 ± 1.1 and 1.9 ± 0.9 d), and time to get-out-of-bed (2.8 ± 0.9 vs 1.8 ± 0.7 and 1.7 ± 0.8 d) as well as a significantly higher VAS score after 24 h of surgery (4.9 ± 1.3 vs 2.5 ± 1.0 and 2.3 ± 0.9) (*P* < 0.05 each) (Tables 3, 4), while there were no significant differences in these variables between the RH group and the LH group (*P*>0.05, Table 4). There was no significant difference in liver function after 24 h of surgery among the three groups (*P*>0.05, Table 3). No postoperative deaths occurred in this study, and 3 patients (5.3%) developed complications, which included gastric retention (*n* = 1) and biliary leakage (*n* = 2) (Table 3). The data for the follow-up visits 3 months after the operations were available for all 57 patients. Four patients (7.0%) had persistent or recurrent preoperative symptoms: 2 patients with abdominal pain, 1 with nausea, and 1 with premature satiety after meals (Table 5).

**Table 2:** Comparison of intraoperative variables in the RH ,LH, and OH Groups

	*RH group*	*LH group*	*OH group*	
	*n = 19*	*n = 13*	*n = 25*	*P value*
Operative time (min)	256.3 ± 57.7	268.4 ± 93.6	190.2 ± 51.8	0.001
Operative time (min) (remove setup time)	216.3 ± 57.7	268.4 ± 93.6	190.2 ± 51.8	0.004
Intraoperative blood loss (ml)	319.5 ± 206.0	476.9 ± 210.8	628.0 ± 231.0	0.000
Rate of blood transfusion	5 (26.3%)	4 (30.8%)	8 (32.0%)	0.916

**Table 3:** Comparison of postoperative variables in the RH ,LH, and OH Groups

	*RH group*	*LH group*	*OH group*	
	*n = 19*	*n = 13*	*n = 25*	*P value*
Postoperative hospital stay(d)	5.5 ± 2.1	4.7 ± 1.7	7.2 ± 2.3	0.002
Time to oral intake(d)	2.2 ± 1.1	1.9 ± 0.9	3.1 ± 1.1	0.002
Time to off-bed activity(d)	1.8 ± 0.7	1.7 ± 0.8	2.8 ± 0.9	<0.001
Liver function				
ALT(U/L)	261.0 ± 164.7	225.4 ± 154.8	305.6 ± 252.9	0.508
AST(U/L)	269.4 ± 162.8	215.8 ± 121.8	271.0 ± 228.8	0.658
ALB(g/L)	35.9 ± 3.1	35.2 ± 7.6	35.4 ± 4.9	0.880
TBil (μmmol/L))	22.3 ± 12.4	17.5 ± 8.3	25.3 ± 16.2	0.245
VAS score	2.5 ± 1.0	2.3 ± 0.9	4.9 ± 1.3	<0.001
Mortality	0 (0%)	0 (0%)	0 (0%)	1.000
Morbidity	1 (5.3%)	1 (7.7%)	1 (4.0%)	0.890

ALT indicates alanine aminotransferase; AST indicates aspartate transaminase; ALB indicates albumin; TBil indicates total bilirubin.

**Table 4:** SNK (Student-Newman-Keuls) *test for operative and postoperative variables in the RH ,LH, and OH Groups

			*RH group*	*LH group*	*OH group*	
			*n = 19*	*n = 13*	*n = 25*	*Significance*
Subset for α = 0.05	Operative time (min)	1			190.1600	1.000
		2	256.2632	268.3846		0.582
	Operative time (min) (remove setup time)	1	216.2632		190.1600	1.000
		2		268.3846		0.239
	Intraoperative blood loss (ml)	1	319.4737			1.000
		2		476.9231		1.000
		3			628.0000	1.000
	Postoperative hospital stay(d)	1			7.2400	1.000
		2	5.5263	4.6923		0.246
	Time to oral intake(d)	1			3.0800	1.000
		2	2.1579	1.9231		0.508
	Time to off-bed activity(d)	1			2.8000	1.000
		2	1.8421	1.6923		0.573
	VAS score	1			4.8800	1.000
		2	2.5236	2.3077		0.565

*Groups are in the same subset indicate no difference between these groups

**Table 5:** Improvement of symptoms after operation

*Symptom*	*Preoperative status*	*Postoperative status*	*P value*
abdominal pain	45 (78.9%)	2 (3.5%)	<0.001
nausea	19 (33.3%)	1 (1.8%)	<0.001
premature satiety after meal	16 (28.1%)	1 (1.8%)	<0.001

## Discussion

Haemangioma is a common benign lesion of the liver. It originates from the mesodermal layer and represents a congenital, non-neoplastic hamartomatous proliferation of vascular endothelial cells [15]. Asymptomatic patients with liver haemangiomas less than 5 cm in diameter require only monitoring through imaging examinations every 6 months or annually to assess the progression of disease [3, 11]. The common indications for surgical treatment in symptomatic patients with liver haemangiomas larger than 5 cm in diameter are pain, rapid growth in size, uncertainty of malignancy, local compression, spontaneous or traumatic rupture, and Kasabach-Merritt syndrome [5, 10]. Since the first resection of liver haemangioma reported by Hermann Pfannenstiel in 1898, the treatments for liver haemangioma have included TAE, enucleation, liver resection, and transplantation [16, 17]. TAE can be used to reduce the size of giant liver haemangiomas and decrease the risk of bleeding during resection. However, vascular recanalization leading to recurrence is common [18–20]. Symptomatic patients with unresectable lesions or multiple haemangiomas are indicated for liver transplantation [21]. Haemangioma is a well-circumscribed, hypervascular and compressible lesion with a clear sheath of compressed liver parenchyma between the haemangiomatous tissues and the normal liver [22]. Enucleation can be performed to remove the liver haemangioma with its surrounding fibrous capsule, which is composed of compressed liver parenchyma. Several authors have reported that enucleation of giant haemangiomas is safer and quicker than liver resection, with better preservation of the liver parenchyma, less morbidity, and less blood loss [6, 7]. On the other hand, Wang et al. [23] concluded that the operative time, blood loss, and blood transfusion requirements for anatomic liver resections were similar to those for enucleation. When a liver haemangioma is giant or when it is at a dangerous anatomical location adjacent to the inferior vena cava or a major hepatic vein, enucleation may cause massive intraoperative blood loss. In such patients, liver resection may be a better approach [24, 25]. In hepatic resection, the FLR volume, SLV and TLV have been used to predict postoperative hepatic dysfunction [26]. Although the safety limit of the FLV remains controversial, several studies have shown that an FLR/TLV ratio of ≤20% is associated with increased complications and a higher likelihood of postoperative hepatic dysfunction in noncirrhotic patients. In our study, the FLR/SLV ratio was between 33.2 and 41.2%, and the resected normal liver volume/resected volume was only between 14.2 and 17.4%. These ratios suggested that there were adequate remnant liver volumes and small losses of normal hepatic parenchyma in our patients. To balance the risk of massive intraoperative bleeding and the preservation of normal hepatic parenchyma, our team prefers to perform hemihepatectomy rather than enucleation for patients without cirrhosis and hepatitis whenever technically possible. To decrease excessive intraoperative blood loss in hemihepatectomy, our team routinely uses selective hemihepatic inflow occlusion for hemihepatectomy and the modified Pringle’s manoeuvre to occlude the inflow of the entire liver when necessary.

The traditional open approach requires a large abdominal incision, which is often associated with a long recovery time. Since the first truly laparoscopic anatomical liver resection in the form of a left lateral sectionectomy was reported in 1996 by Azagra et al., laparoscopic liver resection rapidly progressed and became popular [27]. The main advantages of minimally invasive liver resection over other techniques are its significantly shorter postoperative hospital stay and lower morbidity [28–30]. Robotic surgery is a further development of the minimally invasive technique. The robotic system provides magnified three-dimensional imaging, tremor filtering and motion scaling. Endowrist technology with seven degrees of freedom allows smooth and precise movements that are required in liver resection [31].

The current study aimed to evaluate the clinical efficacy of robotic, laparoscopic, and open hemihepatectomy for giant liver haemangioma carried out by a single team of experienced liver surgeons. Robotic and laparoscopic hemihepatectomies were associated with the following advantages over open hemihepatectomy in our study: less intraoperative blood loss, a shorter postoperative hospital stay, an earlier time to get-out-of bed and earlier oral intake, and a lower VAS score after 24 h of surgery. The modified Pringle’s manoeuvre made the control of bleeding as easy in the RH group and the LH group as in the OH group. The minimal manipulation and the small incision were correlated with less bleeding, a faster postoperative recovery and better pain control. All physicians in our teams were skilled in performing open, robotic and laparoscopic hemihepatectomies. Due to limited two-dimensional vision in laparoscopic surgery, giant liver haemangiomas resulted in limited manipulating space in the LH group, a long setup time in robotic surgery, and a longer operative time in the LH and RH groups. If the setup time in the RH group was excluded, the operative time was significantly shorter in the RH group than in the LH group, while there was no significant difference between the RH and OH groups. The amount of intraoperative blood loss was significantly greater in the LH group than in the RH group. The precise movement and three-dimensional view of the operative field were probably the reasons for the lower amount of bleeding and shorter operation time in robotic hemihepatectomy than in laparoscopic hemihepatectomy. Our study also showed that no significant difference existed among the three groups in terms of the rates of blood transfusion and in the liver function after 24 h of surgery. Yu et al. [32] reported that the levels of ALT and AST after operations in the laparoscopic liver resection group were lower than those in the open liver resection group. Our study also showed no significant differences in postoperative hospital stay, time to oral intake, time to get-out-of bed or VAS scores between the RH and LH groups. Furthermore, most symptoms, such as abdominal pain and nausea, were relieved after hemihepatectomy.

The major limitations of this study are the small sample size and the short duration of follow-up, which may have generated bias in the interpretation of the results. Further multicentre randomized controlled clinical studies with larger sample sizes and longer follow-up periods are needed.

## Conclusion

Robotic and laparoscopic hemihepatectomies were associated with less intraoperative blood loss, less postoperative pain, better postoperative recovery, and a shorter postoperative hospital stay. Compared with laparoscopic surgery, robotic hemihepatectomy was associated with significantly less intraoperative blood loss and a shorter operative time.

## Data Availability

All data generated or analysed during this study are available from the corresponding author on reasonable request.

## Abbreviations

RH: Robotic hemihepatectomy
LH: Laparoscopic hemihepatectomy
OH: Open hemihepatectomy
BSA: Body surface area
FLR: Future liver remnant
SLV: Standard liver volume
TAE: Transarterial embolization
VAS: Visual analogue scale
CT: Computed tomographic
SNK: Student-Newman-Keulss
